# Treatment of Vascular Naevi of Galvanic Cautery

**Published:** 1874-05

**Authors:** B. F. Dawson

**Affiliations:** M. D., etc. New York


### Treatment of Vascular Naevi with the Galvanic Cautery.

BY B. F. DAWSON, M. D., etc. New York.

In vol. iv., No. 3, November, 1871, of this Journal, I published a paper on the "Treatment of Vascular Nævi with the Actual Cautery," and related therein several cases in which the most satisfactory results followed that method of removal, or rather destruction ; and having since operated many times in like manner, I will adhere to the views therein expressed of its advantages and gratifying results.

During the last two years, however, I have had opportunities of witnessing the use of, as well as using myself, the galvanic cautery in various operations, when it is "par excellence" the best means at the command of the surgeon. Having possessed myself of an apparatus, I have used it many times for the destruction of nævi —in some of which other methods, excepting the actual cautery, had proved unsatisfactory—with unfaling success and most gratifying results.

As many surgeons still seem undecided as to the best means for removing this not uncommon nongenital disease, many still adhering to the oldest and most unsatisfactory methods, I deem it not inadvisable fo add my testimony in favor of a method that at least one high authority, Dr. Maas, of Breslau,* pronounces to be followed by the best results, and is much safer than the injection of iron or other coagulating fluid. This opinion he arrived at after having used the galvanic cautery in 112 cases with the following results: *Capillary nævus*—cured, 32 ; improved, 1. *Cavernous or venous nævus*—cured, 72 ; improved, 8 ; died, 3. *Arterial or racemose nævus*—cured, 2 ; improved, 1. *Nævus combined with other tumors*—cured, 6 ; improved, 1 ; result unknown, 2.

The galvanic cautery differs from the actual cautery in the means and facility for heating the needles, while it is superior to the latter from the fact that the degree and duration of the heat is wholly under control of the operator, and consequently it admits of being used with greater care and deliberation, while the actual cautery needles, readily parting with their heat, necessitates their hurried use. These advantages, combined with the admissibility of using very fine needles, are the only advantages the galvanic can claim over the actual, for the effects of the two methods are precisely similar—destruction of the diseased parts by heat. Both methods have the advantage of destroying the nævi in parts of the body where it would be either unsafe or impossible to apply other means, as was the case in the third of the following cases which I have selected as best illustrating the advantages claimed for the galvanic cautery.

CASE I.—Mary O'Neil, one year and eight months, was brought

*Archiv fur Klinische Chirurgie. Vol., XII, 1871.

to me February 17, 1875, with an irregular capillary nævus, the size of a bird's egg, situated immediately beneath the lower left eyelid. The history was as usual—that it was a small spot at birth, but had grown rapidly to its present size, and was a source of annoyance to the parents, as well as disfiguring the child's face. The parents wishing its removal, the following day (18th) I singed it carefully, but thoroughly, with the galvanic cautery, throughout its whole extent, but no deeper than the cutis, so as to guard against unnecessary destruction of tissue, and consequent cicatricial contraction. Cold compresses were then applied and kept in place by a bandage. The next day there was slight consecutive inflammation of the adjacent tissues, very little swelling, and but slight sympathetic congestion of the conjunctivæ. In a week after all signs of congestion had subsided, and a thin scab covered the site of the nævus, which fell off on the twelfth day after the operation, leaving a healthy dark, pink and soft eschar, showing no trace of the nævus, and not in the slightest contrasting or impairing the mobility of the lower lid. Several weeks after, a slight discoloration was the only mark noticeable.

Case II.—Jessie B——, two years old, fine healthy child, was brought to me from Flushing, Nov. 21, 1873, by previous arrangement, to be operated on for a subcutaneous venous nævus situated over the right eyebrow. Compression, collodion and argent. nit. had been used by different physicians without result, as the disease continued to grow to its present size of about half an inch long by one-quarter wide. As in the preceding case, I singed the nævus thoroughly with the platinum needle at a red heat, a wet compress was applied, and the child taken home to Flushing the same afternoon. Five days after, I saw it at my office, and found a firm black scab covering the seat of the nævus. In a few days this scab fell off, leaving a healthy pink cuticle beneath, but at the lower angle a small dark spot showed that a portion of the nævus had escaped destruction. This was destroyed, in like manner, on Dec. 21st, one month after first operation. The result in this case has been perfectly satisfactory, for when seen on Feb. 24th last, the seat of the nævus could only be recognized by a small mark scarcely noticeable, and which the parents have recently informed me is getting fainter each week. In this case I was assisted by my friends, Drs. Rankin, Porter and Hanks.

Case III.—Sarah Hawley, fourteen months old, was brought to me Feb. 21, 1874, at the Dispensary for Sick Children, with a subcutaneous venous nævus in lower portion of the upper right eyelid, and considerably disfiguring the child. The history was one of rapid growth to its present size of a large pea.

The mother stated that she had taken the child to the Eye Infirmary in this city, and that she was advised to have nothing done. On close examination I resolved to operate on the tumor, as from its very rapid growth it was evident that the whole lid would before long be involved, and its function being thus impaired the eye itself would suffer. From the location and deep character of this nævus I could judge of no safe means of removing it except the galvanic cautery. Certainly it would have exposed the eye itself to injury to have attempted its removal by the potential caustics, vaccination, or coagulating injections, for the reason that the effects of these methods would extend beyond the actual site of the nævus, as their action is not wholly under control; the opposite is the case in using the galvanic cautery needle, with which it is possible to destroy slowly and cautiously, and only to the extent deemed safe in view of the consecutive inflamation.

On Feb. 24th, assisted by three of my students, I operated on the case, entering the nævus with the red-hot platinum needle at the lower border of the nævus, which was held by forceps, and thus destroying it subcutaneously by working the point of the needle cautiously to the right and left, avoiding going too deeply. The whole operation was completed within three minutes, and the child on recovering from the chloroform was removed to its home, a wet compress being previously applied.

I saw the child again on the 27th, when a firm scab covered the site of the nævus; there was also some congestion ef the conjunctivæ, but nothing very marked, and but little swelling of the lid. On the 30th I saw the case again and found the scab removed and a slight cicatrix remaining. The eye in all other respects looked healthy. When last seen, April 2d, nothing except a small scar showed where the nævus had been; there was no contraction of the lid, and the mother expressed herself highly pleased at the result. Certainly no better result could have been obtained by other methods of treatment.

These three cases may be considered as fully illustrating the superiority of the galvanic cautery over other means of destroying nævi, and I feel confident that it will before long be universally considered the safest and most reliable means in the majority of cases for removing this so often disfiguring and sometimes dangerous congenital disease.—*American Journal of Obstetrics.*

